# Cefiderocol Retains Antibiofilm Activity in Multidrug-Resistant Gram-Negative Pathogens

**DOI:** 10.1128/AAC.01194-20

**Published:** 2021-01-20

**Authors:** Christine A. Pybus, Christina Felder-Scott, Victor Obuekwe, David E. Greenberg

**Affiliations:** a Department of Internal Medicine, Infectious Diseases and Geographic Medicine, University of Texas Southwestern Medical School, Dallas, Texas, USA; b School of Health Professions, University of Texas Southwestern Medical School, Dallas, Texas, USA; c Department of Microbiology, University of Texas Southwestern Medical School, Dallas, Texas, USA

**Keywords:** cefiderocol, Gram negatives, biofilms, multidrug resistance

## Abstract

Cefiderocol is a siderophore cephalosporin with potent antibacterial activity against a broad range of Gram-negative pathogens, including multidrug-resistant strains. Siderophore antibiotics bind ferric iron and utilize iron transporters to cross the cell membrane. In the biofilm setting, where antibiotic resistance is high but iron scavenging is important, cefiderocol may have advantageous antimicrobial properties. In this study, we compared the antimicrobial activity of cefiderocol to that of seven commonly used antibiotics in well-characterized multidrug-resistant pathogens and then determined their efficacy in the biofilm setting. MIC_90_ values for cefiderocol were consistently lower than those of other antibiotics (ceftolozane-tazobactam, ceftazidime-avibactam, ceftazidime, piperacillin-tazobactam, imipenem, and tobramycin) in all strains tested. Cefiderocol treatment displayed a reduction in the levels of Pseudomonas aeruginosa biofilm (93%, *P* < 0.0001) superior to that seen with the other antibiotics (49% to 82%). Cefiderocol was generally as effective as or superior to the other antibiotics, depending on the pathogen-antibiotic combination, in reducing biofilm in other pathogens. There was a trend toward greater biofilm reduction seen with increased antibiotic dose or with increased frequency of antibiotic treatment. We conclude that cefiderocol effectively reduces biofilm and is a potent inhibitor of planktonic growth across a range of Gram-negative medically important pathogens.

## Abstract (precis)

Cefiderocol is a siderophore cephalosporin with potent antibacterial activity against a broad range of Gram-negative pathogens, including multidrug-resistant strains. Siderophore antibiotics bind ferric iron and utilize iron transporters to cross the cell membrane.

## INTRODUCTION

Gram-negative pathogens worldwide are increasingly developing resistance to antibiotics commonly used to treat infections, including aminoglycosides, carbapenems, cephalosporins, fluoroquinolones, and polymyxins (1–5). Multiple mechanisms exist for microorganisms to evade antibiotic selective pressure. Horizontal gene transfer plays a significant role in multidrug resistance (MDR) (6). Plasmid-encoded extended-spectrum beta-lactamases hydrolyze cephalosporins and carbapenems in the periplasmic space, whereas plasmid-based MCR-1 mediates colistin resistance by cell membrane modification (7–9). In addition, mutations in porins may result in loss of outer membrane permeability to antibiotics, and upregulation and expression of drug efflux pumps can decrease retention of antibiotics (6, 10). Finally, antibiotic resistance is likely not easily lost due to compensatory mutations and horizontal gene transfer (11, 12). Altogether, nosocomial drug resistance is a significant problem that is only becoming worse.

The ability to form biofilm further compounds this problem. Biofilms ultimately increase antibiotic resistance due to a number of factors, including loss of permeability and the presence of slow-growing persister cells (13, 14). Biofilms are clinically relevant, contributing to drug-resistant infections of wounds, prosthetic joints, catheters, the urinary tract, and the lungs (15–18). Thus, new antibacterial agents should be evaluated for their ability to retain activity in the biofilm setting.

To overcome obstacles posed by cell permeability resistance mechanisms, researchers have investigated the use of siderophore-conjugated antibiotics (19, 20). After binding ferric iron, iron transporters (which utilize active transport by their association with outer membrane protein TonB) transfer these antibiotics into the periplasmic space. The antibiotic alters the cell wall (when conjugated to a beta-lactam) or inhibits DNA gyrase in the cytoplasm (when conjugated to ciprofloxacin) (20). Cefiderocol is a siderophore cephalosporin with potent antibacterial activity against a broad range of Gram-negative pathogens, including MDR isolates (21–25). In Pseudomonas aeruginosa, several TonB-driven iron transporters, including PiuA, have been implicated in translocating cefiderocol across the cell membrane (26, 27). Microorganisms forming biofilm may utilize bacterial siderophores to access iron (28, 29). For example, P. aeruginosa requires transport of the siderophore pyoverdine for biofilm formation (29). Consequently, siderophore antibiotics may have unique antimicrobial properties during treatment of biofilm. In this study, we compared the *in vitro* activities of cefiderocol and comparator antibiotics against various MDR strains. We then determined whether cefiderocol can eradicate biofilm in multiple genera and compared its potency to that of other comparator antibiotics.

## RESULTS

Cefiderocol has been previously demonstrated to effectively inhibit growth in a wide range of Gram-negative pathogens (21–25). It has weaker effects on Gram-positive or anaerobic pathogens (24); thus, we limited our study to Gram-negative genera. To assess the efficacy of cefiderocol and comparator antibiotics in inhibiting growth of the MDR strains used for this study, we first determined the MIC in both iron-depleted cation-adjusted Mueller-Hinton broth (ID-CAMHB) (Table 1) and Mueller-Hinton II broth (MHII) (Table 2). Cefiderocol MICs have typically been tested in ID-CAMHB (a CLSI requirement) because the iron-depleted conditions mimic *in vivo* conditions (27). However, iron has been demonstrated to be necessary for mature biofilm formation in diverse bacterial genera (*Pseudomonas*, *Campylobacter*, *Vibrio*, *Serratia*, *Escherichia*, Burkholderia cepacia complex [Bcc]) (30–35). In P. aeruginosa, for example, iron is essential not only for biofilm formation but also for biofilm matrix stability (36). Thus, we compared the fold differences in MIC for all antibiotics used in this study in both ID-CAMHB and MHII. The MIC_90_ of cefiderocol ranged from 0.125 μg/ml (B. cepacia complex) to 1 μg/ml (P. aeruginosa, Acinetobacter baumannii, Escherichia coli) in ID-CAMHB (Table 1). In all strains tested, MIC_90_ values were consistently lower for cefiderocol than for the other agents (for ceftolozane-tazobactam, 16 to >64 μg/ml; for ceftazidime-avibactam, 8 to >64 μg/ml; for ceftazidime, 16 to >64 μg/ml; for piperacillin-tazobactam, ≥64 μg/ml; for imipenem, 16 to >64 μg/ml; for tobramycin, 8 to >64 μg/ml). This trend was also evident for MIC testing in MHII. The cefiderocol MIC_90_ values in MHII were generally 2-fold higher in most cases (Table 2). These results recapitulate the previously observed potency of cefiderocol in inhibiting the planktonic growth of Gram-negative pathogens.

**TABLE 1:**
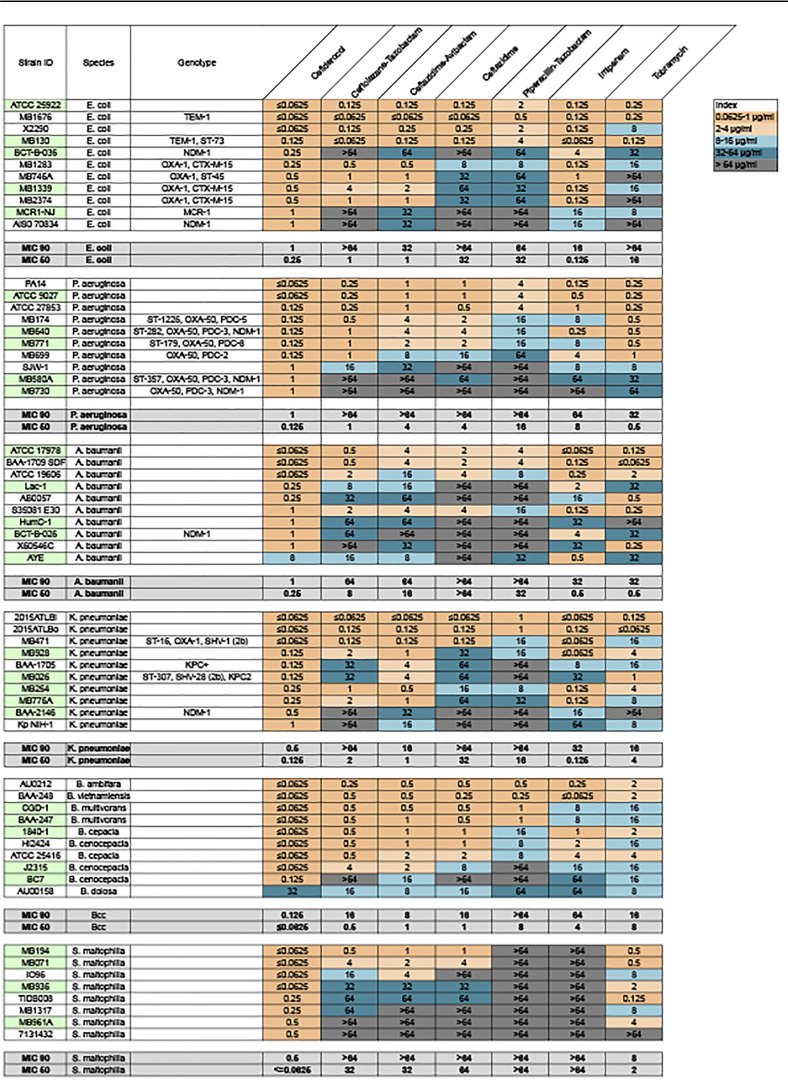
Heat map of MIC for cefiderocol and comparator antibiotics against six MDR Gram-negative genera in MH-CAMHB^*a*^

a Strains used in biofilm assays are highlighted in green.

**TABLE 2:**
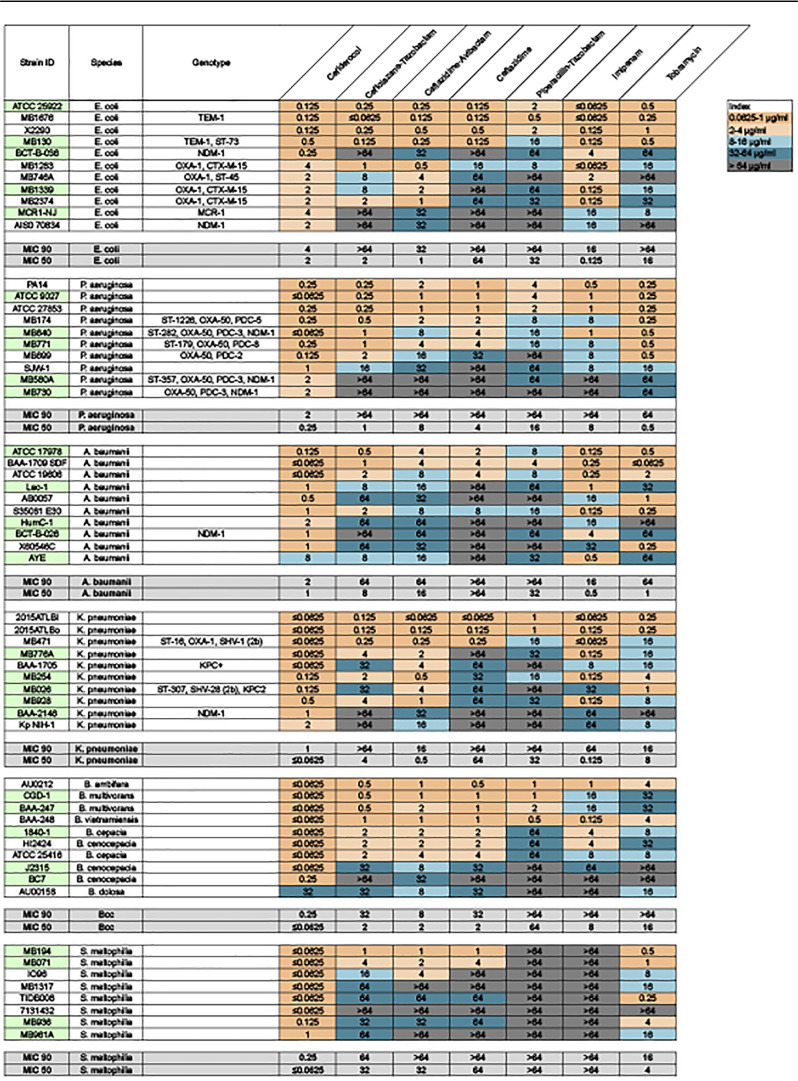
Heat map of MIC for cefiderocol and comparator antibiotics against six MDR Gram-negative genera in MHII^*a*^

a Strains used in biofilm assays are highlighted in green.

**FIG 1:**
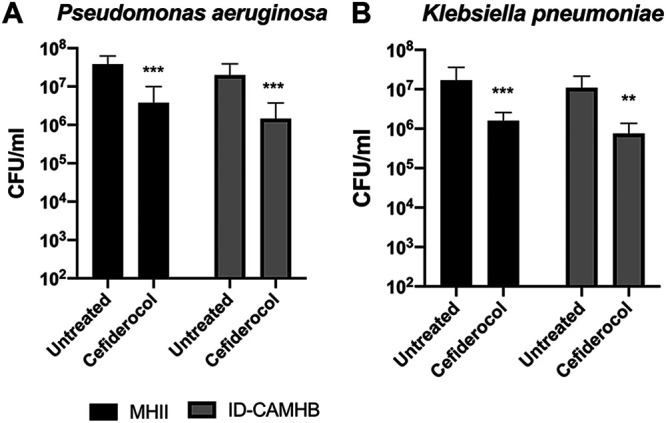
Cefiderocol can reduce existing biofilm in both MHII and ID-CAMHB. Data represent averages of results determined for 4 P. aeruginosa (A) and K. pneumoniae (B) strains in three independent experiments per strain (**, *P* < 0.001; ***, *P* < 0.0001). Error bars represent standard deviations from the means.

Next, we assessed the ability of cefiderocol and comparators to show activity in the biofilm setting. For these experiments, we used MBEC (minimum biofilm eradication concentration) plates, where biofilms previously grown on pegs on the lid of the plate were dosed every 12 h for 24 h with antibiotics and then evaluated for viability. Initially, we compared the biofilm reductions seen under conditions of treatment with antibiotics in ID-CAMHB with those seen with MHII to determine any differences in efficacy due to the limitation or presence of iron, especially for cefiderocol (Fig. 1). Unlike the impact of media on MIC, there were no differences in average biofilm reduction rates for P. aeruginosa or Klebsiella pneumoniae, whether ID-CAMHB or MHII was used in the treatment plate. At 4 μg/ml, there was a >1-log reduction in biofilm levels in both strains compared to untreated controls (*P* < 0.001). Subsequently, further biofilm eradication assays were performed in MHII.

**FIG 2:**
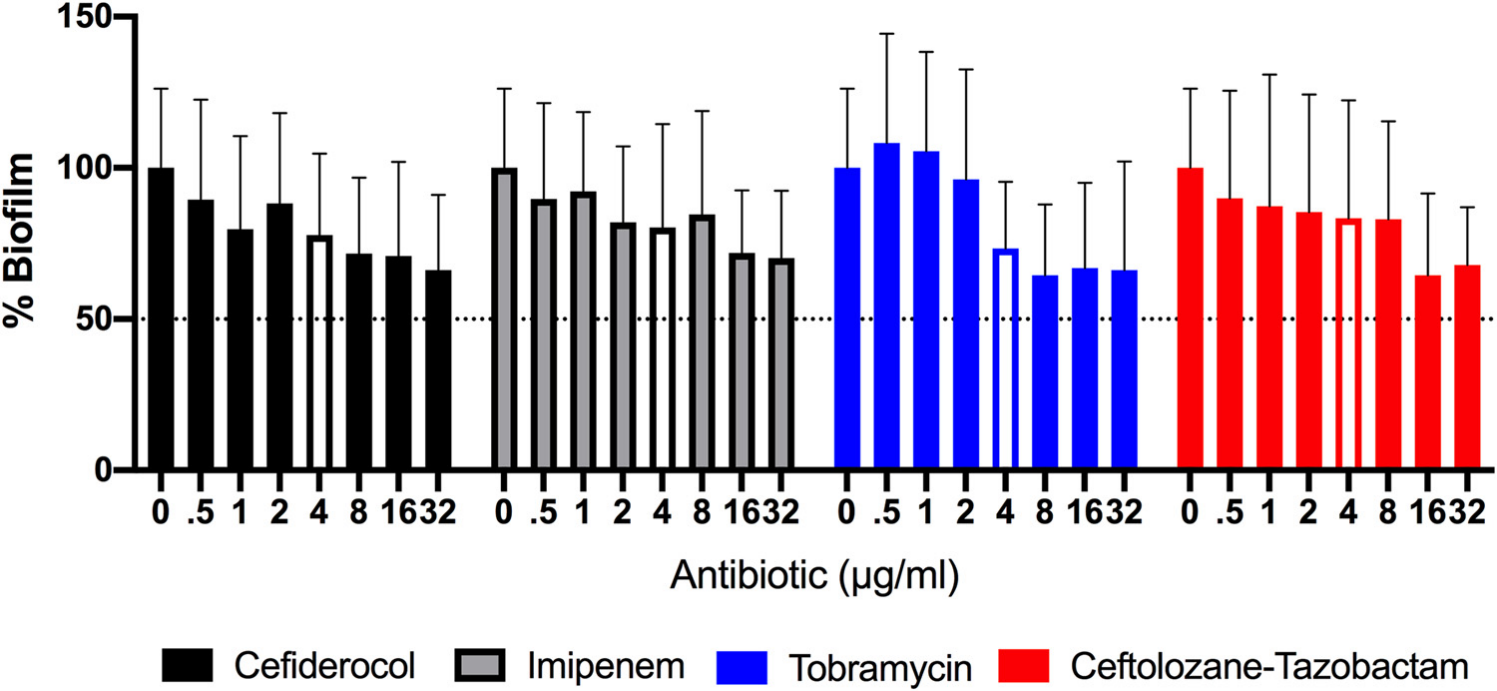
Cefiderocol reduces biofilm biomass in a dose-dependent fashion in P. aeruginosa. Data represent normalized crystal violet assay results for an average of 5 strains with three independent experiments per strain. Concentrations of antibiotics used in eradication assays are indicated with a clear bar.

Four to five strains from each genus tested in the MIC study (with a range of antibiotic sensitivities, highlighted in Table 1) were included for biofilm eradication testing. We first examined dose responses in P. aeruginosa using antibiotic concentrations ranging from 32 to 0.5 μg/ml and a crystal violet assay to measure biofilm biomass (Fig. 2). As measured by total biomass, there was a trend demonstrating dose responses. For cefiderocol, biomass reduction ranged from 20% at an antibiotic concentration of 0.5 μg/ml to 34% at 32 μg/ml. For viability experiments, we utilized a fixed antibiotic concentration of 4 μg/ml.

**FIG 3:**
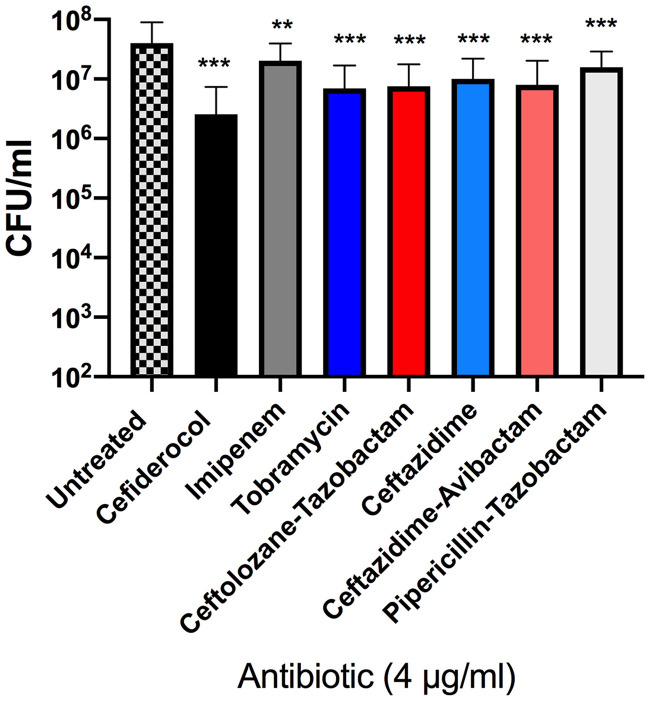
Cefiderocol reduces P. aeruginosa biofilm burden to a greater extent than comparator antibiotics. Data represent viability assay results for an average of 5 strains with three independent experiments per strain. The reduction in biofilm burden associated with each antibiotic was compared to that seen with an untreated control by 2-way ANOVA with Tukey’s multiple-comparison test (**, *P* < 0.001; ***, *P* < 0.0001). Error bars represent standard deviations from the means.

**FIG 4:**
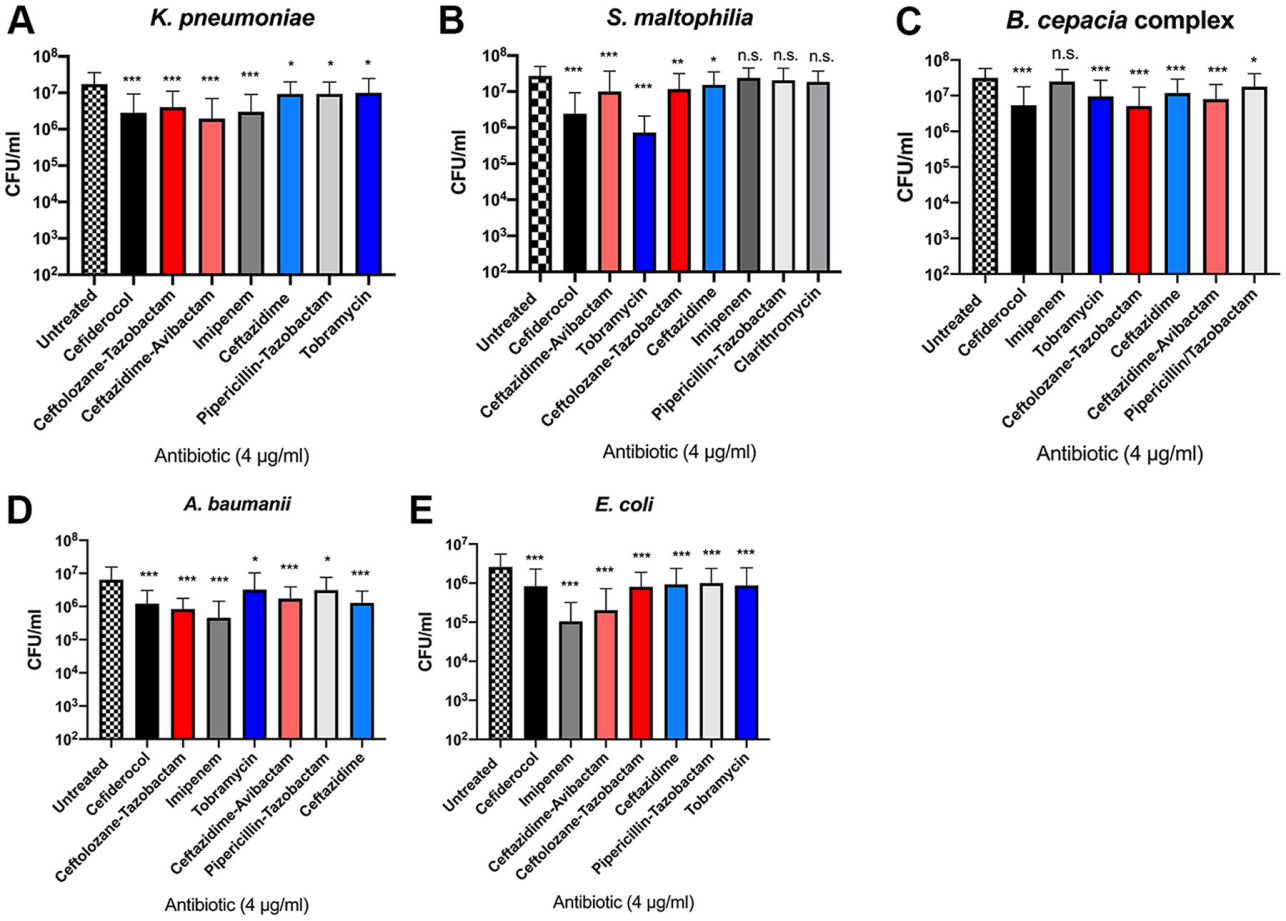
Cefiderocol reduces biofilm in other MDR Gram-negative pathogens. (A) K. pneumoniae. (B) S. maltophilia. (C) B. cepacia complex. (D) A. baumannii. (E) E. coli. Data represent viability assay results for an average of 4 or 5 strains with three independent experiments per strain. The reduction in biofilm burden associated with each antibiotic was compared to that seen with an untreated control by 2-way ANOVA with Tukey’s multiple-comparison test (*, *P* < 0.05; **, *P* < 0.001; ***, *P* < 0.0001). Error bars represent standard deviations from the means.

Based on CFU measurements, cefiderocol treatment displayed a reduction of P. aeruginosa biofilm (>90% reduction compared to the untreated control, or greater than 1 log CFU/ml) that was superior to that seen with the comparator antibiotics. In contrast, imipenem was the least effective (49% reduction) (Fig. 3; see also Table 3). Cefiderocol also reduced biofilm in K. pneumoniae, Stenotrophomonas maltophilia, and B. cepacia complex, by 83% to 91% (Fig. 4; see also Table 3). Cefiderocol was generally as effective at eradication as imipenem in K. pneumoniae or as ceftolozane-tazobactam in B. cepacia complex. For S. maltophilia, tobramycin (99% reduction) and cefiderocol (97% reduction) were superior to the other antibiotics (71% to 87% reduction). In contrast, the most potent antibiotic for A. baumannii and E. coli biofilm reduction was imipenem (>90% reduction versus 67% to 80% reduction reduction with cefiderocol) (Fig. 4; see also Table 3); however, the difference was not statistically significant by two-way analysis of variance (ANOVA) with Tukey’s multiple-comparison test. Taking the results together, cefiderocol reduced biofilm to a degree equivalent to or higher than seen with comparator antibiotics at a dose of 4 μg/ml.

**TABLE 3:** Summary of percent reduction for 24-h assays with 12-h challenge^*a*^

Species	% reduction						
	Cefiderocol	Ceftolozane- tazobactam	Ceftazidime- avibactam	Ceftazidime	Pipericillin- tazobactam	Imipenem	Tobramycin
P. aeruginosa	93.6	81.2	80	74.8	60.7	49.3	82.6
K. pneumoniae	83.7	76.6	88.8	46.9	46.5	82.6	42.7
A. baumannii	80.9	86.9	72.9	79.7	51.5	92.9	48.9
S. maltophilia	97.2	86	87.9	81.9	75.6	71.4	99.1
B. cepacia complex	83.0	83.6	74.6	62.6	43.4	21.3	70.0
E. coli	67.6	68.9	92.2	64.1	61.2	95.9	66.3

a Data represent reduction for biofilms challenged every 12 h for 24 h with cefiderocol and comparator antibiotics in MHII.

To gain some insight into the potency of cefiderocol and other antibiotics in the biofilm setting, we can compare the drug MIC value determined for each bacterial strain, including both drug-sensitive strains and MDR strains, with the percentage of biofilm reduction at a fixed antibiotic concentration (see Table S1 in the supplemental material). In general, for all genera examined, higher levels of biofilm reduction were observed in sensitive strains with lower planktonic MICs. This was consistently observed for cefiderocol treatment in S. maltophilia and B. cepacia complex strains, where MICs were low (1 to ≤0.0625 μg/ml) and biofilm was reduced by 75% to 99%. There were some notable exceptions. For example, although the MIC of cefiderocol in A. baumannii AYE (MDR) was 8 μg/ml, at 4 μg/ml it reduced biofilm better than cephalosporins and better than piperacillin-tazobactam. Further, differences in potency were observed for NDM-1-expressing strains: In E. coli BCT-B-036, no biofilm reduction occurred with 4 μg/ml cefiderocol treatment, although the MIC was 0.25 μg/ml. Similarly, cefiderocol treatment did not effectively reduce K. pneumoniae BAA-2146 biofilm (46%), despite a MIC of 1 μg/ml. On the other hand, for P. aeruginosa (MB640 and MB730) and A. baumannii (BCT-B-026) NDM-1 strains, low cefiderocol MICs (≤0.0625 to 2 μg/ml) were associated with greater (>1 log) biofilm reduction. This study focused on MDR strains, and due to their inherent resistance, a minimum biofilm eradication concentration (MBEC) was difficult to calculate for all comparator antibiotics. MBEC is defined as the lowest concentration of antibiotic required to eradicate the biofilm (37, 38).

**FIG 5:**
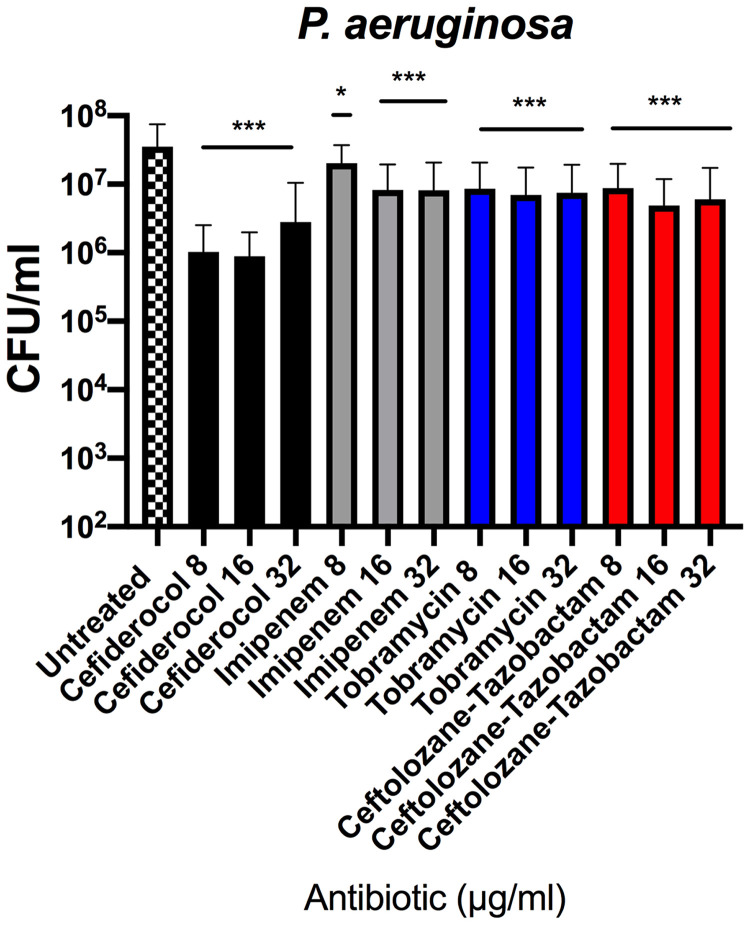
P. aeruginosa biofilm eradication analyzed by viability count does not demonstrate dose dependence. Data represent viability assay results for an average of 5 strains with three independent experiments. The reduction in biofilm burden associated with each antibiotic was compared to that seen with an untreated control by 2-way ANOVA with Tukey’s multiple-comparison test (*, *P* < 0.05; ***, *P* < 0.0001). Error bars represent standard deviations from the means.

We tested whether there was a dose-dependent effect on CFU reduction in biofilm. Increasing doses of cefiderocol, imipenem, tobramycin, or ceftolozane-tazobactam (8, 16, or 32 μg/ml) were administered every 12 h for 24 h in P. aeruginosa, and CFU was determined (Fig. 5). Regardless of the antibiotic used, the efficacy in reducing viable biofilm bacteria plateaued at 16 μg/ml, with no additional CFU decrease seen with higher dosing. To address a potential issue with membrane permeability, we compared the abilities of 8 μg/ml cefiderocol, tobramycin, and ceftolozane-tazobactam to reduce P. aeruginosa strain MB580A biofilm in the presence and absence of 2 μg/ml polymyxin B nonapeptide (PMBN). However, no change in biofilm viability was detected with the addition of PMBN (data not shown).

**FIG 6:**
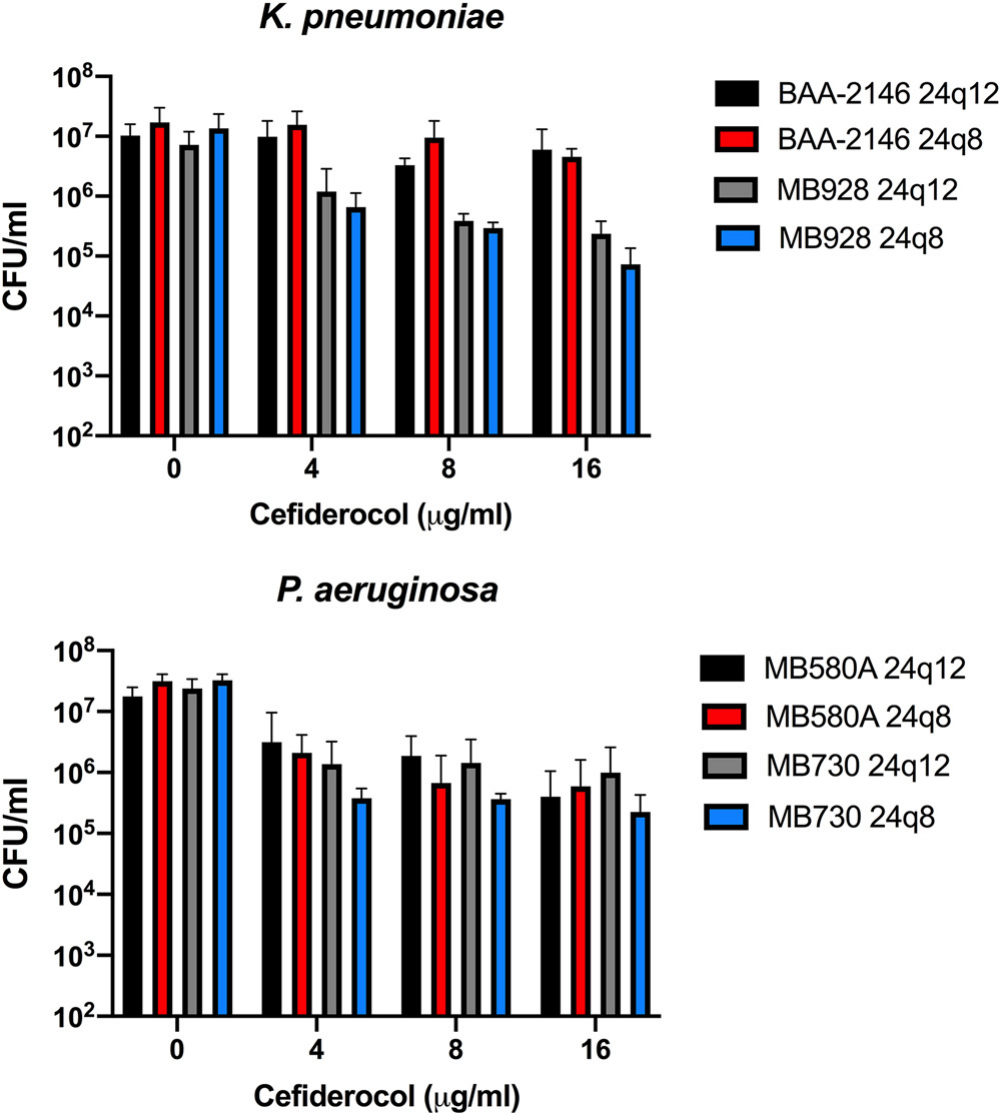
Increasing dosage frequency does not significantly increase biofilm eradication in P. aeruginosa (bottom panel) and K. pneumoniae (top panel). The levels of eradication seen with two MDR isolates for each species were compared by challenging biofilms either every 12 h for 24 h (24q12) or every 8 h for 24 h (24q8). Data represent an average of 3 viability assays. The reductions in biofilm burden seen with the different dosing schedules were compared by 2-way ANOVA with Sidak’s multiple-comparison test, but no significance was found. Error bars represent standard deviations from the means.

We next tested whether dosing frequency altered biofilm breakdown. We compared breakdown levels in two MDR strains of P. aeruginosa (MB580A and MB730) and of K. pneumoniae (BAA-2146 and MB9228) using a schedule of dosing either every 12 h for 24 h or every 8 h for 24 h. Additionally, we examined the impact of increases in dosing frequency with increasing dosage amount using 4, 8, and 16 μg/ml cefiderocol. The results of these assays are illustrated in Fig. 6. Biofilm reductions were compared for each dosing regimen at each concentration of cefiderocol using 2-way ANOVA with Sidak’s multiple-comparison test. Although no significant difference was found, there was a trend to further biofilm reduction with dosing every 8 h (q8) versus dosing every 12 h (q12) (Table S2). As a negative control, we examined cefiderocol’s ability to reduce Gram-positive biofilm grown and treated every 8 h for 24 h (24q8) in iron-limited media (ID-CAMHB). We observed no differences between treated and untreated Staphylococcus aureus biofilm (see Fig. S1 in the supplemental material).

Given that we were interested in determining cefiderocol activity in MDR isolates, the doses used for the biofilm reduction assays were frequently below the MICs for comparator antibiotics in these strains. We therefore examined biofilm reduction potency in sensitive strains that had similar planktonic MICs of both cefiderocol and comparator antibiotics. For these experiments, we utilized 24q8 dosing and CFU determinations. Biofilms were grown in either MHII or ID-CAMHB and then treated in the same respective media (Fig. S2). For the individual isolates tested, the potency of cefiderocol in CFU reduction was no different from or was less than that seen with the two comparator antibiotics, imipenem and tobramycin.

## DISCUSSION

This study demonstrated that cefiderocol reduces biofilm in MDR Gram-negative bacteria. At a fixed dose, it was superior to comparator antibiotics in *Pseudomonas* and superior to most comparators in the other tested MDR pathogens. In the planktonic setting, cefiderocol was superior to comparator antibiotics in inhibiting bacterial growth, in both antibiotic-sensitive and MDR strains, as has been previously reported (21–25).

In addition, there were trends that demonstrated both concentration-dependent and time-dependent eradication of biofilm with cefiderocol. First, we observed that increased cefiderocol doses further reduced biofilm but that the results plateaued at a dose of 16 μg/ml. Comparator antibiotics imipenem, tobramycin, and ceftolozane-tazobactam showed similar results. The antibiotics assayed have two different mechanisms of action; tobramycin prevents protein synthesis, whereas imipenem, ceftolozane-tazobactam, and cefiderocol interfere with cell wall synthesis. Furthermore, their mechanisms of cell entry are likely different; tobramycin may enter the cell through the OprB porin or by means of other active transporters (39), while cefiderocol appears to utilize TonB-dependent iron transporters (26, 27). Consequently, the mechanism responsible for the plateauing of the antibiotic doses used does not appear to correspond to an interrupted transporter or mutation of the target site. It is possible that exopolymeric substances inhibit a certain amount of antibiotic diffusion through the biofilm to the cell membrane or that the effective dosage of antibiotic is reduced when it encounters the nonviable cells that constitute part of the biofilm matrix or that antibiotics are effluxed. Second, while not statistically significant, the results seen after increasing the treatment time and dosage in two MDR strains (P. aeruginosa MB730 and K. pneumoniae MB928) showed a trend toward greater biofilm reduction. Factors affecting these results may include biofilm penetration, for reasons discussed above. Pharmacokinetic/pharmacodynamic studies showed a favorable outcome when cefiderocol was administered on a q8h dosing schedule in animal models and in renally unimpaired patients (40–42). Future work will include a larger array of isolates to test what the optimal dosing and concentration parameters should be to target biofilm in MDR or sensitive strains.

The study was designed to assess whether cefiderocol was active in the biofilm setting in MDR strains. Because of this, the concentration used for the biofilm reduction studies was frequently below the MIC for the comparator antibiotics. This is a limitation of the study in terms of addressing the issue of whether cefiderocol activity is related to its planktonic potency or whether its mechanism of entry into the cell provides an added advantage in the biofilm state. Early testing seems to support the former hypothesis as cefiderocol was not superior to imipenem or tobramycin when tested in strains that were more sensitive to these comparators. In addition, whether the biofilm was grown under iron-limited or iron-replete conditions did not make a significant difference for most strain-antibiotic combinations. A broader look at activity in multiple sensitive strains and under different biofilm conditions is ongoing. Since biofilm formation promotes siderophore production (43), we speculate that siderophore transporters may be upregulated in the biofilm setting. Future transcriptional profiling and colocalization studies could elucidate whether cefiderocol shows improved uptake through the siderophore pathway in the biofilm setting and whether it is retained in the bacterial cell. In addition, the ability of cefiderocol to have antibiofilm activity in *in vivo* infection models is yet to be determined. In summary, cefiderocol retains activity in the biofilm setting, including in isolates that are otherwise resistant to comparator antibiotics.

## MATERIALS AND METHODS

### Bacterial strains and growth conditions.

Clinical isolates of MDR Escherichia coli, Pseudomonas aeruginosa, Acinetobacter baumannii, Klebsiella pneumoniae, Burkholderia cepacia complex (Bcc), and Stenotrophomonas maltophilia were included for study. Isolates were obtained from the American Type Culture Collection or were clinical isolates obtained from Samuel Shelburne (MD Anderson), John LiPuma (University of Michigan), and Joanna Goldberg (Emory). Isolates came from a variety of sources (blood, urine, sputum) and hosts (e.g., malignancy, cystic fibrosis). Strains were maintained as cryofrozen stocks and incubated on Remel tryptic soy sheep blood agar (Thermo Fisher Scientific) at 37°C in 5% CO_2_ for 18 to 24 h before testing in MIC assays. For biofilm assays, a single colony from a blood agar plate was inoculated into cation-adjusted Mueller-Hinton II (MHII) broth and incubated with shaking overnight. The following morning, the culture was diluted into Dulbecco’s phosphate-buffered saline (PBS) and measured by the use of a spectrophotometer (optical density at 600 nm [OD_600_], 0.07 to 0.08) to achieve a cell density of 1 × 10^8^ CFU/ml. This PBS stock was further diluted into MHII to achieve a starting inoculum of 5 × 10^5^ CFU/ml.

Comparator antibiotics included ceftolozane-tazobactam, ceftazidime-avibactam, ceftazidime, piperacillin-tazobactam, imipenem, and tobramycin. Cefiderocol was obtained from Shionogi & Co., Ltd., Japan. Comparator antibiotics were obtained from the University of Texas Southwestern Medical Center campus pharmacy.

### MIC.

MICs were determined for each strain in triplicate utilizing the Clinical and Laboratory Standards Institute (CLSI) broth microdilution method (CLSI 2015) for cefiderocol and seven comparator antibiotics in both iron-depleted cation-adjusted Mueller-Hinton broth (ID-CAMHB; obtained from International Health Management Associates, Inc., Schaumberg, IL, or from Thermo Fisher Scientific) and MHII. E. coli ATCC 25922 and P. aeruginosa ATCC 9027 strains were assayed regularly as controls. The acceptable range for cefiderocol in ID-CAMHB was 0.06 to 0.5 μg/ml (44).

### Minimum biofilm eradication concentration (MBEC) assays.

Biofilm was grown in MBEC plates (Innovotech, Alberta [AB], Canada). Bacteria were inoculated with 5 × 10^5^ CFU/ml bacteria in MHII (or Luria-Bertani broth for E. coli) and incubated with shaking at 37°C for 24 h. Afterward, the lid of the plate (with pegs containing biofilm) was transferred to a fresh 96-well plate with antibiotics in MHII, LB, or ID-CAMHB and incubated as before. A second dose was administered 12 h later by moving the pegs to a new plate (stored at 4°C) with or without the antibiotic. At 48 h, the lid was washed in PBS and then either fixed and stained with crystal violet or sonicated to determine viable cell numbers. Assays comparing an 8-h dosing regimen over 24 h (24q8) to a 12-h dosing regimen over 24 h (24q12) were processed similarly, except that fresh challenge plates were not refrigerated for either test.

### Crystal violet assay.

Biofilm on the pegs was fixed with methanol and air-dried. Pegs were stained with crystal violet solution (45) (solubilized in acetic acid) for 20 min. OD_570_ was measured in a Synergy Biotek plate reader.

### Viability assay.

Each peg was broken from the plate in a sterile fashion with pliers and added to 1 ml PBS in a 14-ml Falcon tube. The tubes were sonicated in a water bath for 15 min. After vortex mixing was performed, CFU levels were measured by drip-plating serial dilutions on sheep blood agar.

## Supplementary Material

Supplemental file 1
