# Supplementary material for: Cefiderocol Retains Antibiofilm Activity in Multidrug-Resistant Gram-Negative Pathogens
Source: Antimicrob Agents Chemother. 2021 Jan 20;65(2):e01194-20. doi: 10.1128/AAC.01194-20 (PMC7849010; doi:10.1128/AAC.01194-20)
Supplement: Supplemental file 1 [file AAC.01194-20-s0001.pdf]

P. aeruginosa

Strain ID	Cefiderocol		Ceftolazane-Tazobactam		Ceftazidime-Avibactam		Ceftazidime		Pipericillin-Tazobactam		Imipenem		Tobramycin	
	MIC	% R	MIC	% R	MIC	% R	MIC	% R	MIC	% R	MIC	% R	MIC	% R
ATCC 9027	≤0.0625	92.4	0.25	86.3	1	94.7	1	91.8	4	78	1	38.7	0.25	95.7
MB640	≤0.0625	98.9	1	99.0	8	96.8	4	99.2	16	77.6	1	99.7	0.5	97.3
MB771	0.25	95.8	1	98.2	4	98.4	4	96.6	16	66.5	8	47.3	0.5	96.7
MB580A	2	72.8	>64	11	>64	0	>64	6.4	64	16.4	>64	0	64	14.9
MB730	2	94.8	>64	37.4	>64	0	>64	0	>64	7.3	>64	0	64	37.8

K. pneumoniae

	Cefiderocol		Ceftolazane-Tazobactam		Ceftazidime-Avibactam		Ceftazidime		Pipericillin-Tazobactam		Imipenem		Tobramycin	
	MIC	% R	MIC	% R	MIC	% R	MIC	% R	MIC	% R	MIC	% R	MIC	% R
BAA - 2146	1	46.1	>64	61.2	32	46.3	>64	63.5	>64	72.5	64	39.6	>64	57.9
MB928	0.5	91.3	4	98.5	1	15.6	64	45.4	32	30.1	0.125	95.1	8	99.3
MB026	0.125	91.1	32	3.5	4	40.0	64	0	>64	99.1	32	8.6	1	88.9
MB254	0.125	96.6	2	99.9	0.5	49.2	32	50.6	16	0	0.125	98.6	4	99.9
MB776A	≤0.0625	97.6	4	99.7	2	57.7	>64	42.6	32	66.9	0.125	98.0	16	99.9

S. maltophilia

Strain ID	Cefiderocol		Ceftolazane-Tazobactam		Ceftazidime-Avibactam		Ceftazidime		Pipericillin-Tazobactam		Imipenem		Tobramycin	
	MIC	% R	MIC	% R	MIC	% R	MIC	% R	MIC	% R	MIC	% R	MIC	% R
MB194	≤0.0625	99.4	1	98.9	1	21.9	1	96.1	>64	16.1	>64	99.6	0.5	99.99
MB071	≤0.0625	99.96	4	99.2	2	0	4	41.4	>64	12.2	>64	99.8	1	99.997
MB936	0.125	99.2	32	6.9	32	31.2	64	0	>64	24.3	>64	60.6	4	83.2
MB961A	1	75.6	64	11.2	>64	21.0	>64	29.1	>64	32.2	>64	10.0	16	98.5

B. cepacia complex

Strain ID	Cefiderocol		Ceftolazane-Tazobactam		Ceftazidime-Avibactam		Ceftazidime		Pipericillin-Tazobactam		Imipenem		Tobramycin	
	MIC	% R	MIC	% R	MIC	% R	MIC	% R	MIC	% R	MIC	% R	MIC	% R
BAA-247	≤0.0625	84.0	0.5	97.5	2	83.6	1	90.2	2	40.2	16	45.9	32	69.0
BC7	0.25	90.9	>64	77.5	32	61.9	>64	34.9	>64	47.2	>64	25.4	>64	60.0
CGD-1	≤0.0625	99.6	0.5	99.0	1	99.1	1	99.0	1	95.1	16	53.4	32	96.3
J2315	≤0.0625	75.0	32	46.2	8	65.5	32	46.2	>64	27.1	64	0	>64	65.6
1840-1	≤0.0625	93.3	2	76.6	2	94.7	2	76.6	64	76.6	4	77.1	8	99.2

A. baumanii

Strain ID	Cefiderocol		Ceftolazane-Tazobactam		Ceftazidime-Avibactam		Ceftazidime		Pipericillin-Tazobactam		Imipenem		Tobramycin	
	MIC	% R	MIC	% R	MIC	% R	MIC	% R	MIC	% R	MIC	% R	MIC	% R
17978	0.125	99.2	0.5	99.9	4	60.6	2	88.2	8	95.5	0.125	96.6	0.5	99.3
AYE	8	72.0	8	0	16	41.1	>64	42.5	32	46.4	0.5	75.7	64	98.1
HumC-1	2	27.4	64	29.2	64	30.7	>64	51.9	>64	62.1	16	67.8	>64	63.6
Lac-1	1	49.4	8	80.4	16	30.4	>64	69.6	64	70.6	1	0	32	83.7
BCT-B-026	1	97.6	>64	0	64	66.6	>64	76.7	64	78.9	4	96.2	64	82.9

E. coli

Strain ID	Cefiderocol		Ceftolazane-Tazobactam		Ceftazidime-Avibactam		Ceftazidime		Pipericillin-Tazobactam		Imipenem		Tobramycin	
	MIC	% R	MIC	% R	MIC	% R	MIC	% R	MIC	% R	MIC	% R	MIC	% R
BCT-B-036	0.25	0	>64	0	32	0	>64	16.8	64	62.3	4	83.9	64	0
25922	0.125	99.0	0.25	91.6	0.25	94.2	0.125	96.7	2	94.2	≤0.0625	99.7	0.5	97.1
Mcr1_NJ	4	3.5	>64	57.9	32	62.4	>64	73.0	>64	73.6	16	69.1	8	48.9
MB1339	2	60.1	8	61.4	2	54.8	>64	41.4	64	39.6	0.125	99.9	16	99.7
MB130	0.5	99.5	0.125	79.9	0.25	58.7	0.125	83.7	16	94.9	0.125	99.96	0.5	99.0

Supplemental Table 1. Comparison of MIC and percent biofilm reduction in MHII for biofilms challenged every 12h for 24h. Percent reduction is shown for individual strains.

P. aeruginosa

Strain ID	MIC	Percent reduction with Cefiderocol 24q12			Percent reduction with Cefiderocol 24q8		
		4 µg / ml	8 µg / ml	16 µg / ml	4 µg / ml	8 µg / ml	16 µg / ml
MB580A	2	82.2	89.3	97.7	93.3	97.9	98.1
MB730	2	94.3	94.0	95.8	98.8	98.9	99.3

K. pneumoniae

Strain ID	MIC	Percent reduction with Cefiderocol 24q12			Percent reduction with Cefiderocol 24q8		
		4 µg / ml	8 µg / ml	16 µg / ml	4 µg / ml	8 µg / ml	16 µg / ml
BAA-2146	1	3.6	68.0	41.5	8.8	44.1	73.4
MB928	0.5	83.3	94.6	96.7	95.2	97.8	99.5

Supplemental Table 2. Comparison of MIC and Percent Biofilm Reduction in MHII for biofilms challenged every 12 h for 24 h versus every 8 h for 24 h. Percent reduction is shown for individual strains.

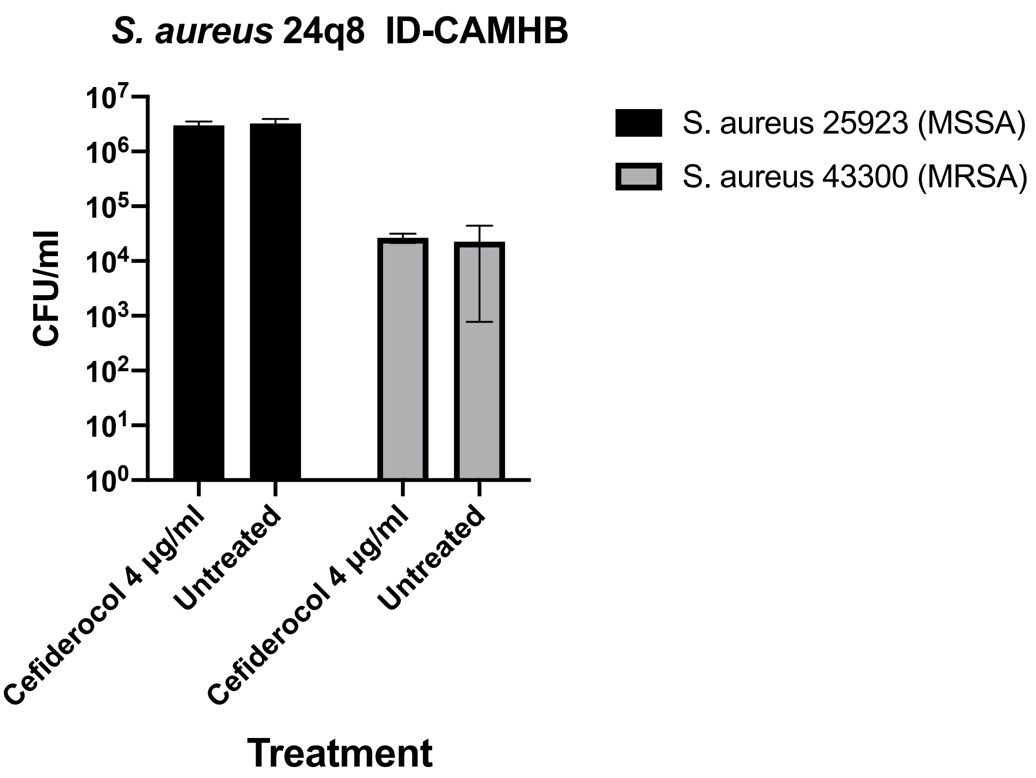

Supplemental Figure 1. Cefiderocol does not reduce Gram-positive biofilm (negative control). Biofilms in ID-CAMHB were treated every 8 h for 24 h. Assays were repeated twice. Error bars represent standard deviation from the mean.

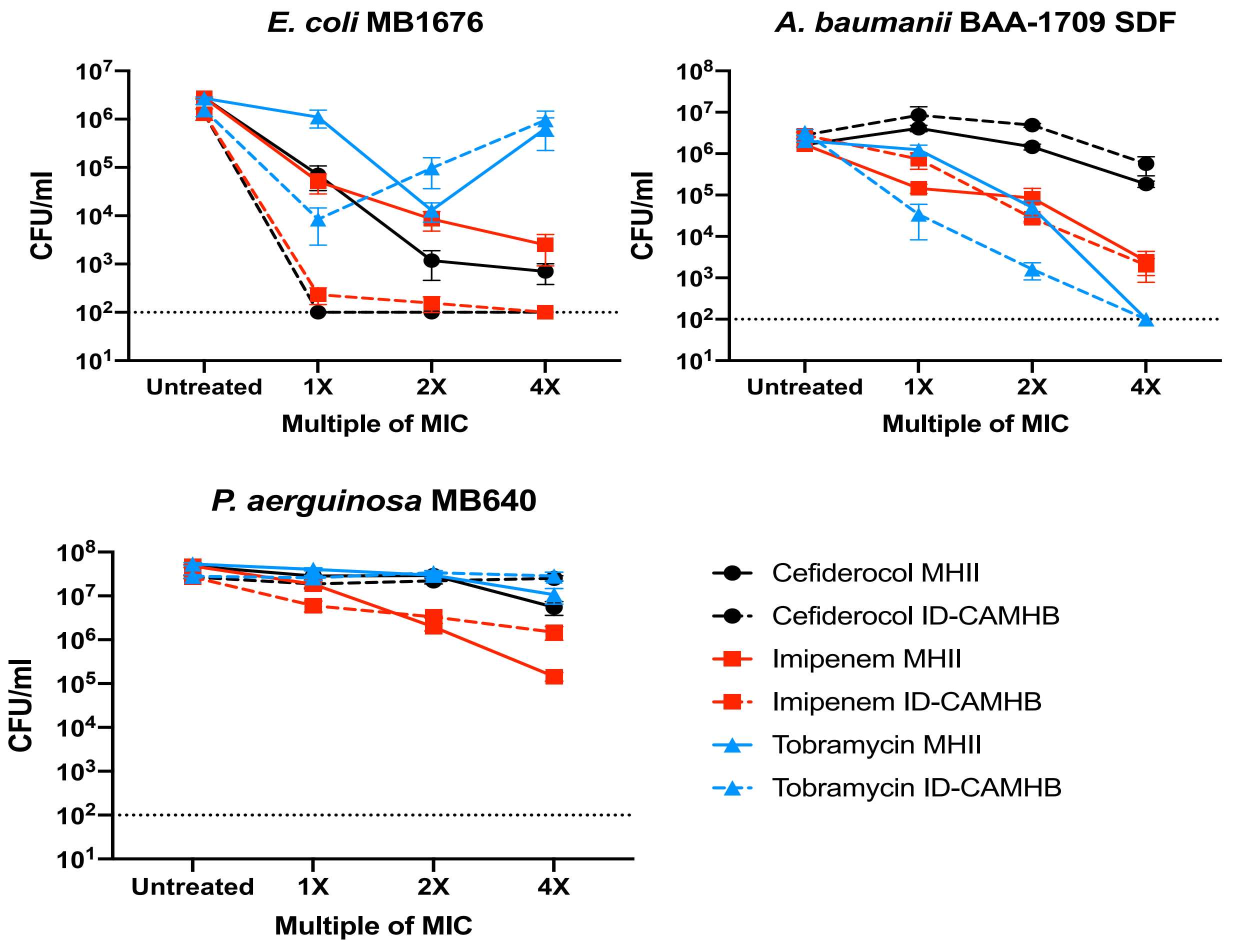

Supplemental Figure 2. Biofilm reduction in sensitive strains. Eradication for three sensitive isolates was compared by challenging biofilms every 8 h for 24 h (24q8) in either MHII (solid line) or ID-CAMHB (dashed line). Data represents an average of 3-4 viability assays. Line at the bottom of the graph represents limit of detection. Error bars represent standard error of the mean.
